# Differential Expression of Proteins Associated with the Hair Follicle Cycle - Proteomics and Bioinformatics Analyses

**DOI:** 10.1371/journal.pone.0146791

**Published:** 2016-01-11

**Authors:** Lei Wang, Wenrong Xu, Lei Cao, Tian Tian, Mifang Yang, Zhongming Li, Fengfeng Ping, Weixin Fan

**Affiliations:** 1 Department of Dermatology, Nanjing Medical University Affiliated Wuxi People’s Hospital, Wuxi, Jiangsu, China; 2 Department of Dermatology, Nanjing Medical University Affiliated Wuxi Second Hospital, Wuxi, Jiangsu, China; 3 Department of Neurobiology, Nanjing Medical University, Nanjing, Jiangsu, China; 4 Jiangsu Key Laboratory of Oral Diseases, Nanjing Medical University, Nanjing, Jiangsu, China; 5 Department of Clinical Laboratory Science, Nanjing Medical University Affiliated Wuxi People’s Hospital, Wuxi, Jiangsu, China; 6 Department of Dermatology, the First Affiliated Hospital of Nanjing Medical University, Nanjing, Jiangsu, China; University Hospital Hamburg-Eppendorf, GERMANY

## Abstract

Hair follicle cycling can be divided into the following three stages: anagen, catagen, and telogen. The molecular signals that orchestrate the follicular transition between phases are still unknown. To better understand the detailed protein networks controlling this process, proteomics and bioinformatics analyses were performed to construct comparative protein profiles of mouse skin at specific time points (0, 8, and 20 days). Ninety-five differentially expressed protein spots were identified by MALDI-TOF/TOF as 44 proteins, which were found to change during hair follicle cycle transition. Proteomics analysis revealed that these changes in protein expression are involved in Ca^2+^-regulated biological processes, migration, and regulation of signal transduction, among other processes. Subsequently, three proteins were selected to validate the reliability of expression patterns using western blotting. Cluster analysis revealed three expression patterns, and each pattern correlated with specific cell processes that occur during the hair cycle. Furthermore, bioinformatics analysis indicated that the differentially expressed proteins impacted multiple biological networks, after which detailed functional analyses were performed. Taken together, the above data may provide insight into the three stages of mouse hair follicle morphogenesis and provide a solid basis for potential therapeutic molecular targets for this hair disease.

## Introduction

The hair follicle (HF) is a regenerating system that undergoes a cyclic process of growth, regression and resting phases (i.e., anagen, catagen, and telogen). At the same time, the HF and skin exhibit circadian rhythms in gene transcription and protein expression. HFs are composed of dermal and epidermal compartments that exist in a complex system. Ralf Paus proposed a comprehensive guide for the recognition and classification of distinct stages of hair follicle morphogenesis[1]. The hair follicle has been widely used as an easy and effective model for tissue regeneration and hair biology research. On the basis of this model, researchers have found induction and maintenance efforts that involve platelet-derived growth factor (PDGF) isoforms in the anagen phase of mouse hair follicles[2]. In addition, when overexpressed in the skin, Wnt10b can induce a switch in the hair-follicle phase from telogen to anagen[3]. Another study demonstrated that bone marrow mesenchymal stem cells (BM-MSCs) in Wnt1a-conditioned medium can activate dermal papilla (DP) cells and promote hair follicle regrowth[4]. Recently, some plant extracts were used to treat alopecia[5]. If the hair follicle cycle is interrupted, hair follicle-related diseases such as androgenetic alopecia might occur. Therefore, research regarding the regulation of the hair follicle cycle is urgently needed. To unveil the molecular mechanisms involved in the hair follicle cycle, proteomics and bioinformatics analyses, both of which are subfields of systems biology aimed at a system-level understanding of biological processes, were performed. Proteomics has been highlighted as a technique that focuses on the simultaneous study of the expression of the vast majority of proteins in a cell or organ under different experimental conditions. Bioinformatics is another means of providing a system-level analysis of biological networks using computational tools. This proteomics strategy has been applied in a variety of studies. For instance, this approach has been used to evaluate the impact of taxol on DP cells[6]. Furthermore, heat shock protein 70 and mitochondrial ribosomal protein S7, two proteins involved in the aggregative property of cultured DP cells, were identified by this method[7]. Using the shotgun proteomics technique and network analysis, ITGB1, IGFBP3, and THBS1 were selected as possible hair-growth-modulating protein biomarkers[8]. However, HFs cannot only be studied as individual components, as these components do not function in isolation in living organisms. From a systems biology point of view, HF cycling involves structural and temporal complexities. The present study involves the mouse skin hair cycle at specific time points (0, 8, and 20 days), which is more suitable for obtaining sufficient amounts of proteins than using pure cultured DP cells. Time-course gene- and protein-expression profiling of mouse skin has identified novel candidates involved in hair-cycle regulation. Additionally, experiments using mouse skin at different stages (anagen, catagen, telogen) can assess interfering factors from the microenvironments of tissues and other types of cells. The microenvironment influences the ability of hair periodic growth. A full hair cycle is not exhibited *in vitro* due to the absence of neural, extrafollicular tissue and vascular or endocrine signals.

Based on the aforementioned experimental advantages and biological characteristics, the present study combines proteomics, cluster analysis and bioinformatics analysis to investigate protein expression and biological networks during the hair cycle.

## Materials and Methods

### Animals

Syngenic C57/BL6 mice (females, 6–8 weeks of age, 15–18 g), all in the telogen stage of the hair cycle, were obtained from the Model Animal Research Center of Nanjing University (Nanjing, China)[9] and maintained in a controlled environment with 12-hour light/dark periods and free access to mouse chow and water. The care and use of all animals in this article strictly adhered to the local animal protection laws of China and were approved by the Experimental Animal Ethics Committee at the Southeast University (permit number 20140301150). All methods were carried out in accordance with approved guidelines, and all efforts were made to minimize suffering.

### Hair cycle induction and skin harvest

Anagen was introduced into the dorsal skin of mice in the telogen phase of hair cycle by applying a 1:1 mixture of melted wax and rosin after injected intraperitoneally with 240mg/kg Avertin (2, 2, 2-Tribromoethanol, Sigma-Aldrich, T48402) [10–13]. After ensurance of adequate anesthesia and the mixture hardening, the wax/rosin mixture was peeled off the skin to pluck all telogen hair shafts, as this method can yield homogeneous populations of anagen follicles that are morphologically indistinguishable from those occurring spontaneously. At 0, 8, and 20 days after depilation, Avertin was administrated by intraperitoneal injection. Once adequate anesthesia was ensured, the mice were sacrificed by cervical dislocation, and every effort was made to minimize suffering. The dorsal skin of the mice was harvested perpendicular to the paravertebral line as described previously[14] and immediately frozen in liquid nitrogen for further analysis.

### Protein extraction

Skin samples of mice from three independent groups were obtained at the aforementioned time points. First, the skin samples were homogenized in lysis buffer (7 M urea, 2 M thiourea, 4% [w/v] CHAPS, 2% [w/v] DTT, and 2% [v/v] IPG buffer [pH 3–10]) in the presence of 1% w/v protease inhibitor cocktail (Pierce Biotechnology, Rockford, IL). Next, the mixture was shaken at 4°C for 1 h, and insoluble molecules were removed by centrifugation at 40000×g, 4°C for 1 h. The protein concentration in each sample was determined using the Bradford method with BSA as the standard.

### Two-dimensional electrophoresis (2-DE)

Protein samples (120 μg) were loaded onto 24-cm, pH 3–10 IPG strips (Bio-Rad Amersham Bioscience, Uppsala, Sweden). Next, the IPG strips were rehydrated in rehydration solution. Following isoelectric focusing, the IPG strips were equilibrated, run on an Ettan DALT 12 electrophoresis system (GE Healthcare, San Francisco, CA, USA), and visualized by silver staining[15–17].

### Statistical analysis

As previously described [17], we stained the gels, and ImageMasterTM 2D Platinum Software (Version 5.0, Amersham Bioscience,Swiss Institute of Bioinformatics, Geneva, Switzerland) was used for spot detection, quantification, and comparative analyses. Expression level was determined by the relative volume of each spot in the gel and expressed as %Volume (%Vol = [spot volume/∑volumes of all spots resolved in the gel]). To reflect the quantitative variations in the protein spot volumes, we normalized the spot volumes as a percentage of the total volume of all the spots present in a gel as previously described[16]. The values obtained for 9 experiments were pooled for calculation of the mean and standard derivations. Protein spots differentially changed across time-points were determined whether the *p* <0.05 (one-way ANOVA).

### Protein identification by MALDI-TOF/TOF

Differential protein spots were excised from the stained gel, dehydrated in acetonitrile (ACN), and dried at room temperature[17]. The proteins were reduced using 10 mM DTT/25 mM NH_4_HCO_3_ at 56°C for 1 h, after which they were alkylated *in situ* with 55 mM iodoacetamide/25 mM NH_4_HCO_3_ in the dark at room temperature for 45 min. Subsequently, the gel fragments were washed with 50% ACN, dried in a SpeedVac, and then rehydrated with 2–3 μl trypsin (Promega, Madison, WI, USA) solution (10 ng/μl in 25 mM NH_4_HCO_3_) at 4°C for 30 min. The excess liquid was discarded, and the gel plugs were incubated at 37°C for 12 h. A final concentration of 0.1% trifluoroacetic acid (TFA) was added to arrest the digestive reaction.

Spotting was achieved by pipetting 1 μl of the analyte onto the MALDI target plate in duplicate and subsequently adding 0.05 μl of 2 mg/ml CHCA in 0.1% TFA/33% ACN containing 2 mM (NH_4_)_3_PO_4_. The Bruker peptide calibration mixture (Bruker Daltonics) was also spotted for external calibration. All samples were then analyzed on a time-of-flight Ultraflex II mass spectrometer (Bruker Daltonics) in the positive-ion reflectron mode.

Each acquired mass spectrum was processed using Flex-Analysis v2.4 and Biotools 3.0 software packages (Bruker Daltonics). The MS/MS spectra were cross-referenced with the IPI rat database, employing MASCOT (v2.4) in the automated mode. Peptide masses were assumed to be monoisotopic masses, and cystines were assumed to be iodoacetamides.

The peptide mass tolerance was set to 100 ppm, and the maximum of missed cleavage sites was set to 1. Positive identification was achieved only when a 100-ppm mass accuracy met with significant probability, and nearly all dominant signals of the spectrum were assigned to the identified protein. The calculated mass and pI were compared and further evaluated.

### Cluster analysis

For every protein spot identified, the mean abundance values from three repeats were calculated and normalized. Cluster 3.0 software was used to process the normalized abundance values, and the protein spots were clustered using a *k-*means algorithm with the similarity metric of Euclidian distance. Only the sample with the least number of clusters and sufficient separation of expression patterns across time was selected. The results were displayed using TreeView software.

### Bioinformatics analysis

The identified proteins were classified algorithmically based upon evolutionary relationships to obtain a functional regulatory network[18]. The differentially expressed proteins overlaid on the network were assembled using Pathway Studio (v5.00) software (Ariadne Genomics, MD, USA), and the identified cellular process was confirmed via the Pub Med/Medline hyperlink embedded in each node.

#### Gene ontology (GO)

A GO enrichment analysis with DAVID v6.7 was performed to provide a functional annotation of an extensive list of genes derived from our proteomics study, which was conducted with the default settings. Statistically significant differences (*p*<0.05) were identified using Fisher’s exact test [19].

### Western blot analysis

The protein levels of Lamin A/C, Vimentin, and Annexin A1 in mouse skin were analyzed at three aforementioned time points. Antibodies against Lamin A/C (2032, diluted 1:1000; cell signaling technology), Vimentin (3932, diluted 1:1000; cell signaling technology), Annexin A1 (3299, diluted 1:1000; Cell Signaling Technology), and β-actin (ab6276, diluted 1:1000; Abcam) were used as internal controls.

## Results

### Identification of specific time points in the mouse hair cycle

**Fig 1:**
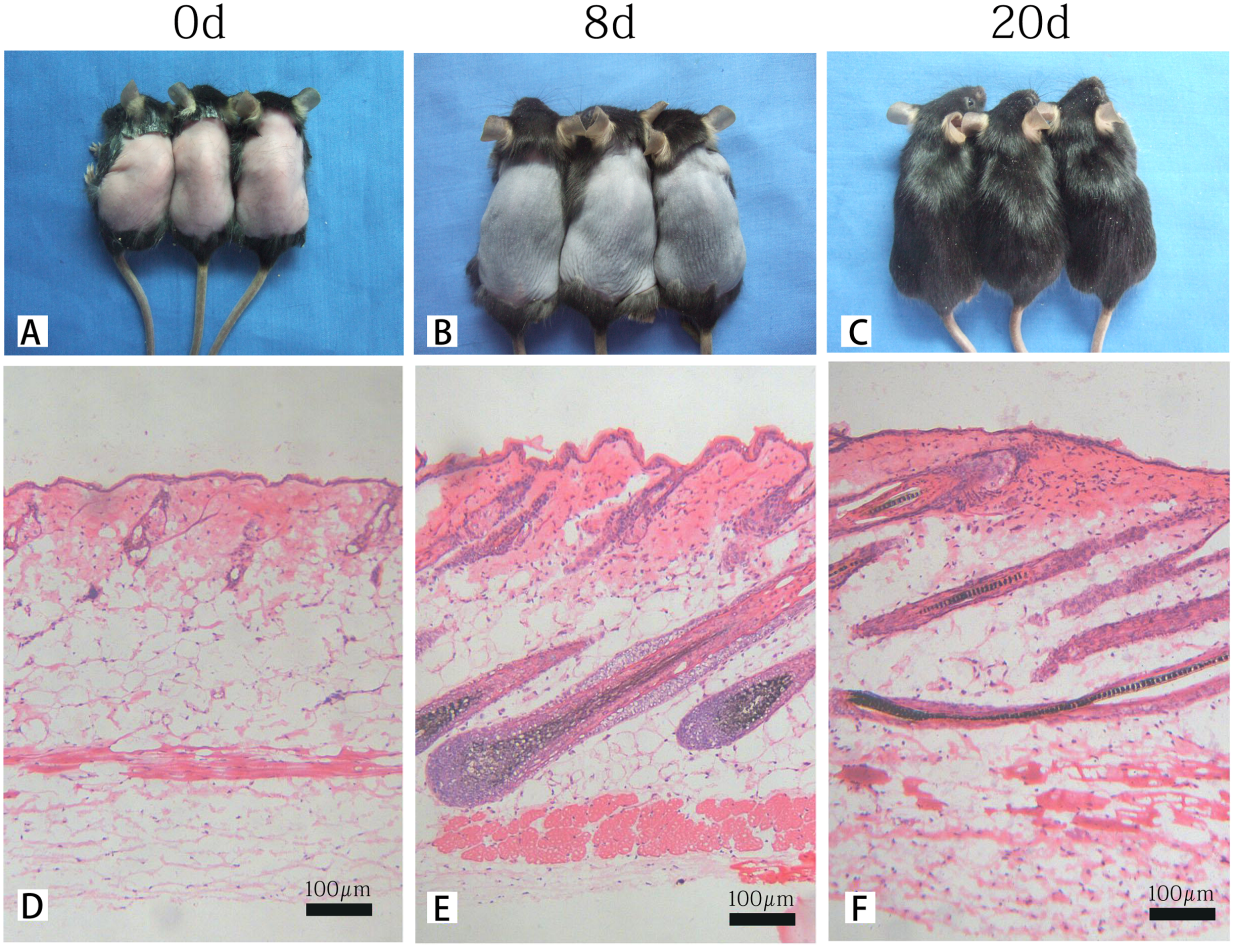
Representative images of skin color, hair regrowth and hematoxylin–eosin (HE) staining of skin samples. (A) telogen (0 d, pink); (B) anagen (8 d, black); and (C) catagen (20 d); (D-F) Stage-specific morphology of distinct HF compartments, skin thickness, and hair follicle percentage. Scale bar: 100 μm. (D) entire hair follicle resides in the dermis. (E) hair bulb in the subcutis. Tip of the hair shaft emerges through the epidermis. (F) narrower bulb and shorter hair follicle than those in anagen.

As shown in Fig 1 and consistent with a previous report [11], we confirmed three time points (days 0, 8, and 20) that were representative of the telogen, anagen, and catagen stages, respectively. After depilation, mouse dorsal skin and hair regrowth were photographed on days 0, 8, and 20. Telogen, anagen, and catagen stages were observed by microscopy in longitudinal sections of the dorsal skin using hematoxylin-eosin (HE) staining.

### Identification of proteins related to the hair cycle

2-DE was performed on the day 0, 8, and 20 skin samples over a pH range of 3–10. Image MasterTM 2D Platinum software revealed significant differences (*P<*0.05) between any two time points for 95 of the observed spots. Forty-four different proteins were identified via MALDI-TOF/TOF and SwissProt searches. The characteristics and functions of the proteins thus identified are listed in S1 and S2 Tables.

### Cluster analysis

**Fig 2:**
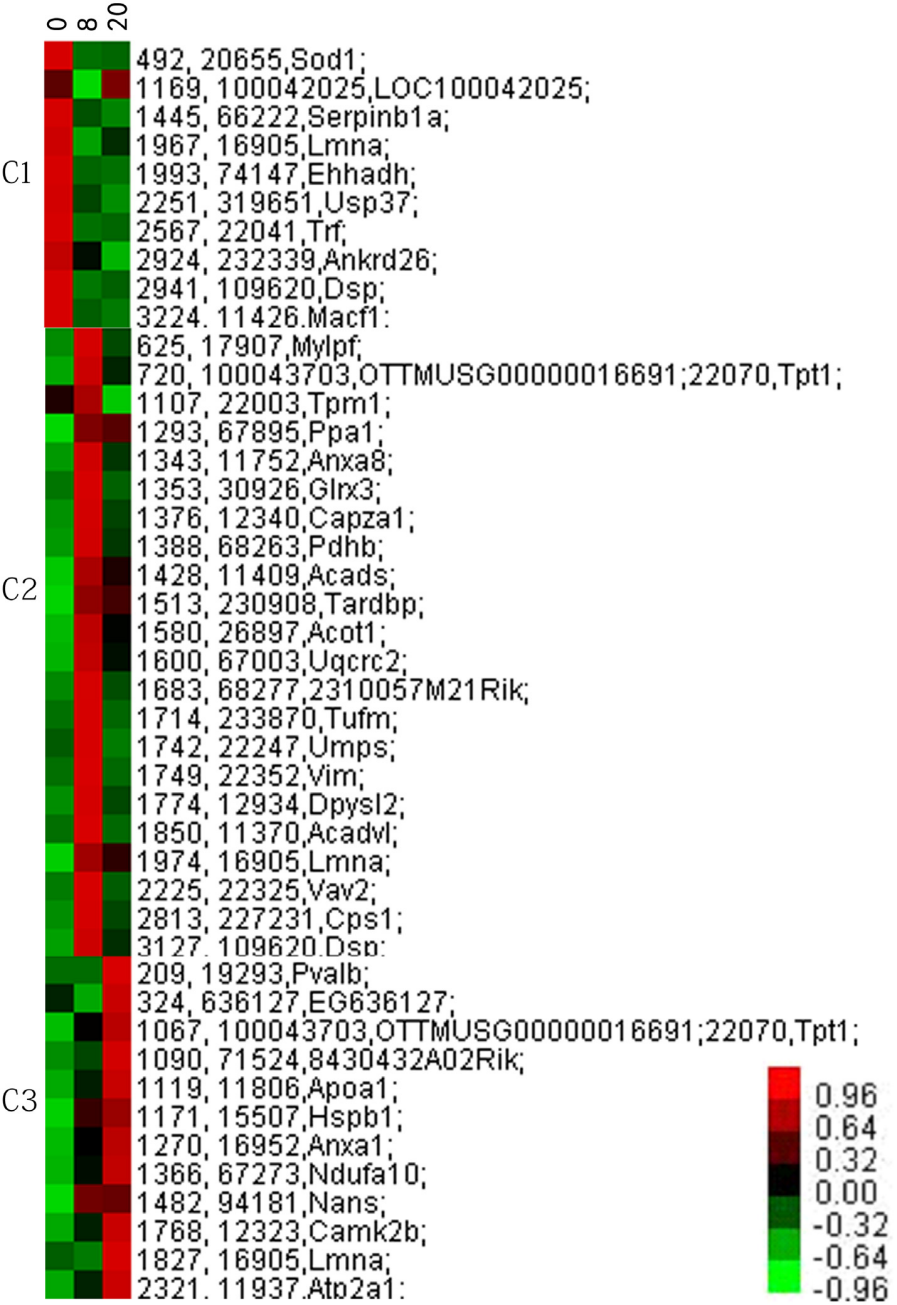
Cluster analysis of the expression levels of 44 differentially expressed protein spots in the three phases. Three clusters (C1, C2, C3) corresponding to three distinct expression patterns were identified. Green represents down-regulated expression, whereas red indicates up-regulated levels.

Cluster analysis was performed to further characterize the specific and unique expression patterns of the identified protein spots; the proteins were finally grouped into three clusters (Fig 2). The three unique expression patterns included high expression levels at the telogen (day 0, Fig 2, C1), and increased expression levels at anagen (day 8, Fig 2, C2), and catagen phases (day 20, Fig 2, C3).

### Bioinformatics

**Fig 3:**
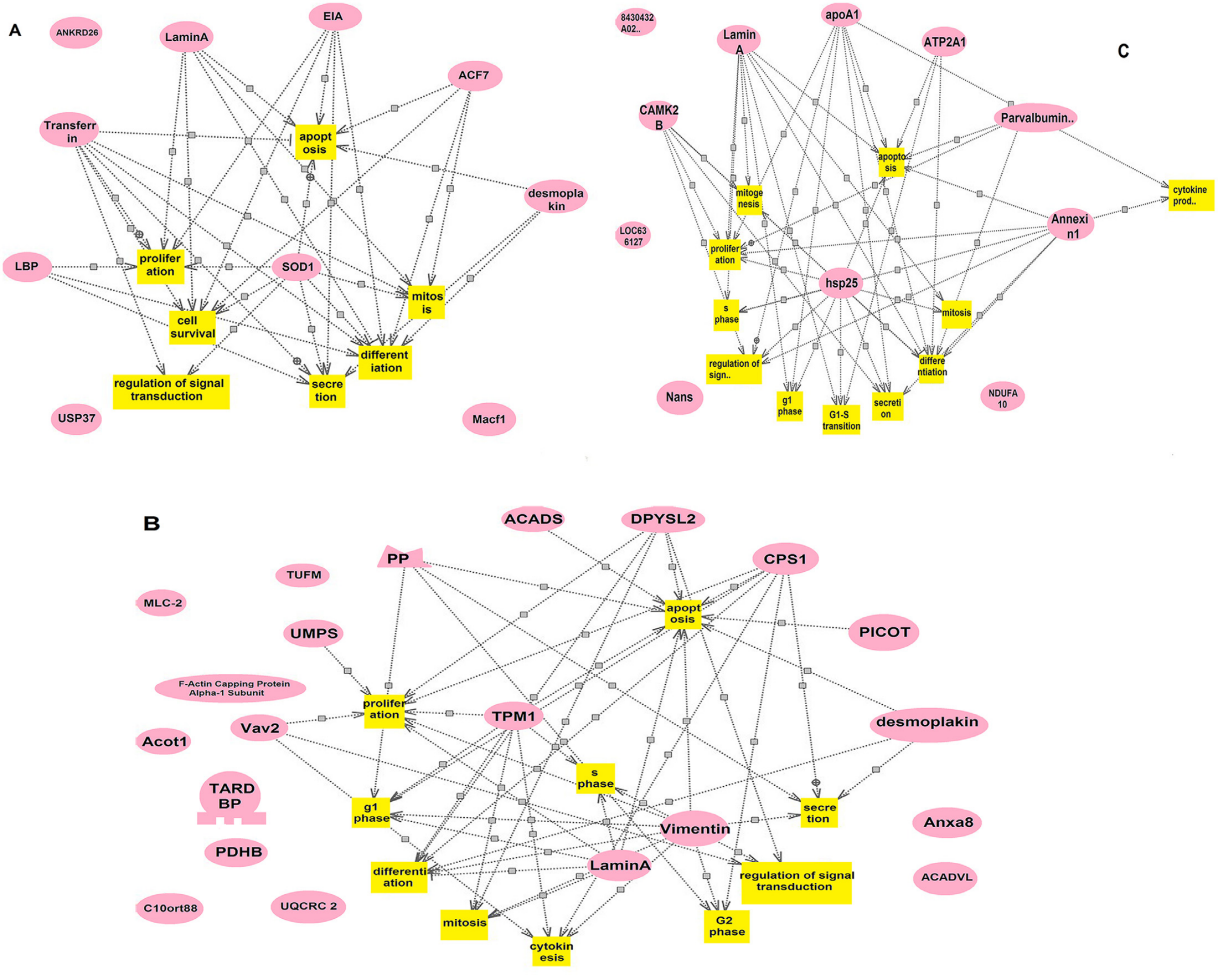
Potential biological network of differentially expressed proteins during hair cycling. Cellular processes (yellow squares) involved in each cluster and all relevant proteins (red ovals) validated by literates (the line). Regulation events are displayed with arrows. (A) The cellular processes of high expressed proteins in telogen involved in C1; (B) The cellular processes of high expressed proteins in anagen involved in C2; (C) The cellular processes of high expressed proteins in catagen involved in C3.

Pathway Studio software was used to estimate the networks of differentially expressed proteins in mouse skin. Relevant proteins are shown as red ovals, and cellular processes are represented by yellow squares (Fig 3). Regulation events are displayed with arrows. At least 6 different biological network clusters can be observed in apoptosis, proliferation/cell survival, regulation of signal transduction, cell phase transition, mitosis/cytokinesis, and secretion.

#### GO enrichment analysis

**Fig 4:**
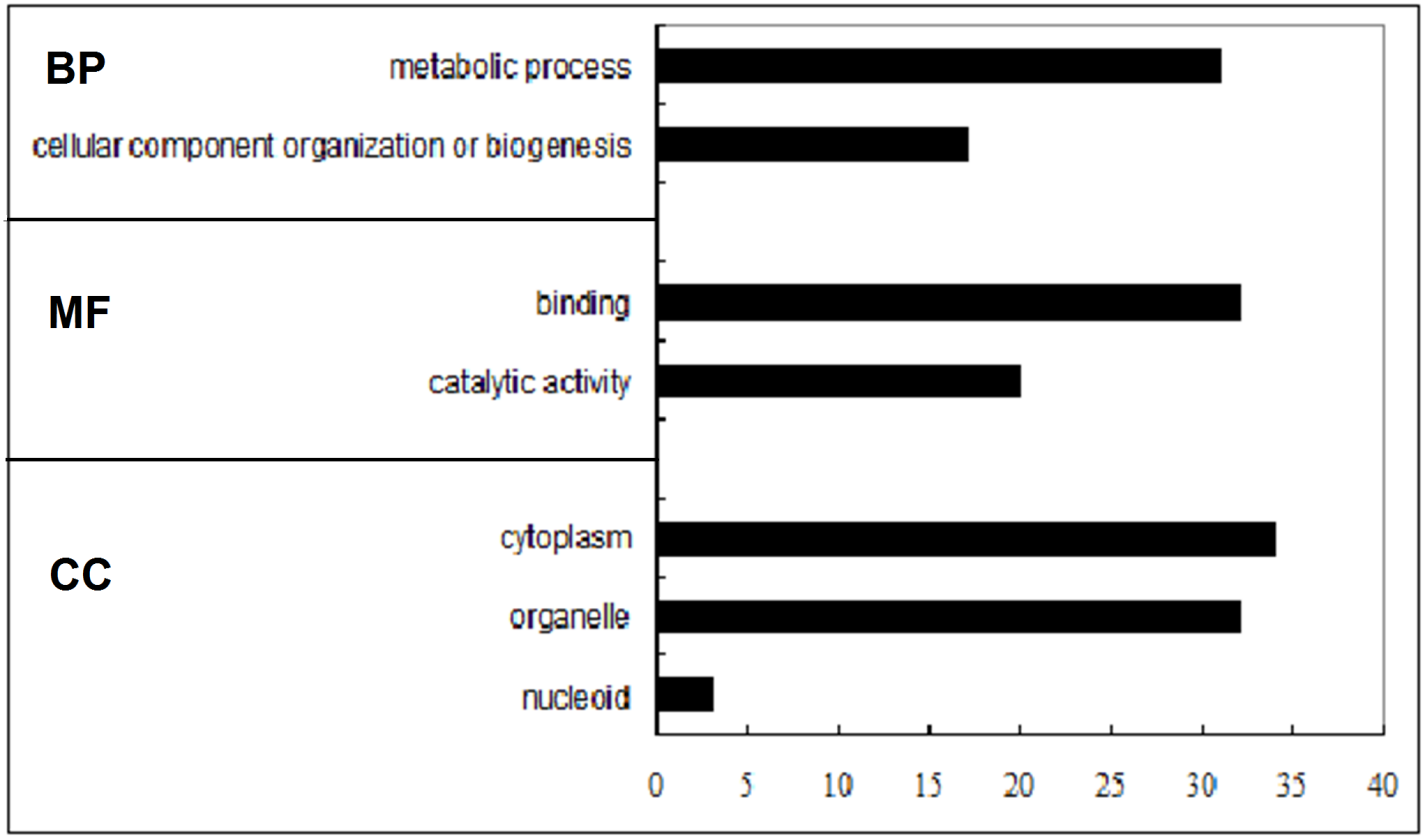
The GO enrichment analysis performed by the DAVID bioinformatics resources online. The results were grouped based on the biological process (BP), molecular function (MF) and cellular component (CC). Statistically significant differences (p-value <0.05) were determined using Fisher’s exact test.

The results were grouped based on the biological process (BP), cellular component (CC) and molecular function (MF). Among the 44 proteins that were differentially expressed, 31 (70.5%) were enriched in the metabolic process (i.e., lipid, phosphorus, oxoacid metabolic process, etc.), 17 (38.7%) were involved in the cellular component organization or biogenesis. 32 (72.7%) were responsible for molecular binding, 20 (45.5%) were identified for catalytic activity. 34 (77.3%) were assigned to cytoplasm, 3 (6.8%) to nucleoid, and 32 (72.7%) to organelle. (Fig 4 and S2 Table)

### Western blot analysis

**Fig 5:**
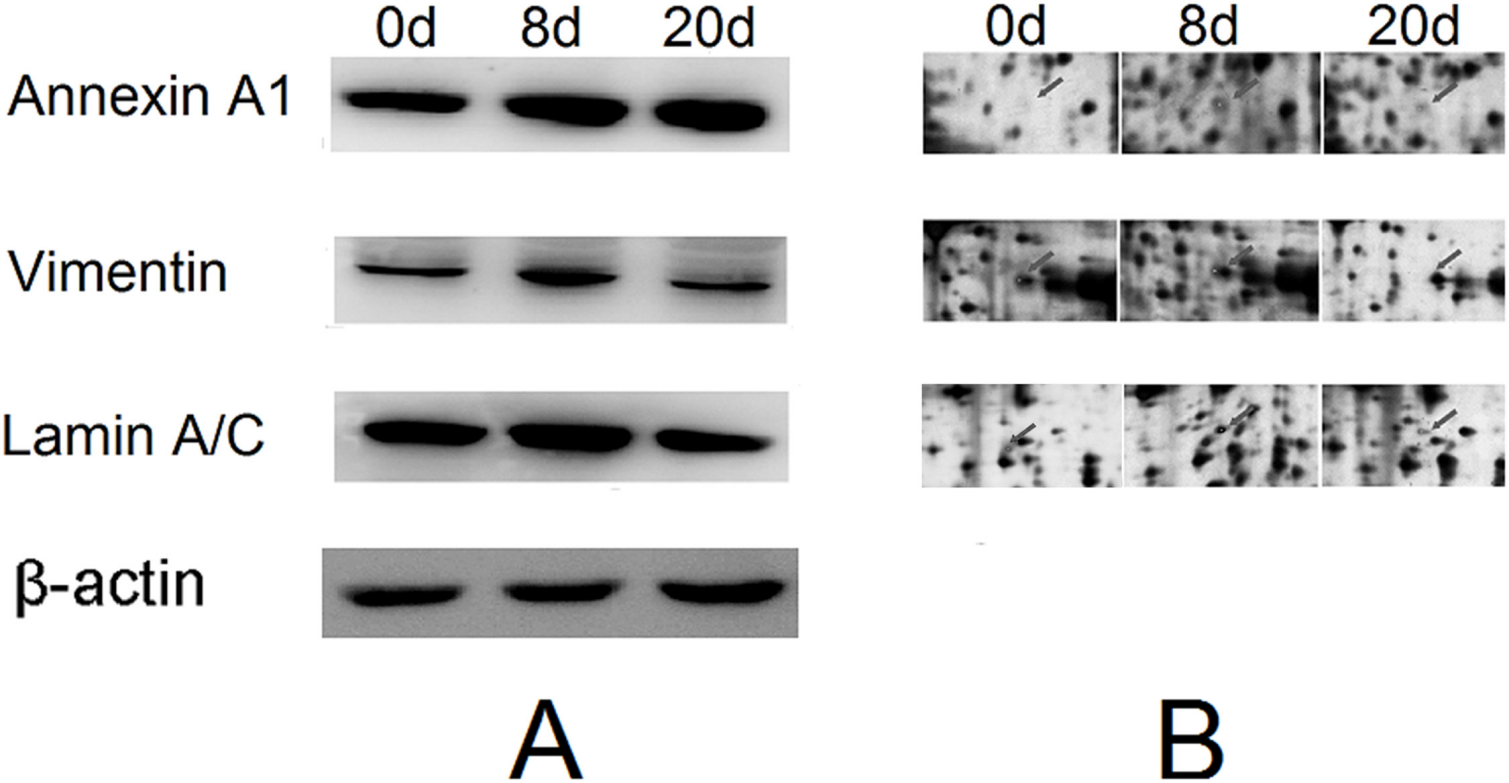
Protein expression confirmation by western blot. (A) Left panel: Results of Western blot analysis performed with specific protein antibodies and protein extracts from mouse skin. (B) Right panel: Magnified spot images of the same molecular weights distributed in 2-DE gels.

Western blot analyses were performed to verify the expression determined for the 3 proteins under consideration (Annexin A1, Vimentin, Lamin A/C), which were selected from S1 Table. The protein expression changes observed in the immunoblots were consistent with the 2-DE results (Fig 5).

## Discussion

The HF is an essentially autonomous mini-organ that is easily accessible. After initial formation and a prolonged period of growth, the HF undergoes cycles of destruction and regeneration throughout life[11]. These cycles involve three periods: growth (anagen), regression (catagen), and rest (telogen). The precise nature, timing, and intersection of the inductive and regulatory signals implicated in formation and growth requires further investigation. In the present study, the C57BL/6 mouse was a suitable model to evaluate the hair cycle. C57BL/6 mice have no melanocytes in the skin, and melanogenesis is observed only in hair follicles. Furthermore, melanogenesis is strongly related to the growth stage of the hair cycle. Thus, melanin is produced only in the anagen phase (black skin) and ceases production at the telogen phase (pink skin) (Fig 1)[9, 20, 21]. Briefly, the time points at days 0, 8, and 20 represent the stages of telogen, anagen, and catagen, respectively, in depilation-induced HF cycles[11].

In fact, this mouse study cannot be extrapolated to explain the behavior of human HFs. However, a similar mechanism may exist between follicular units during the HF cycle[22].

Based on the above model, the proteomics and bioinformatics data clearly confirm the impacts of the differentially expressed proteins examined herein on the biological networks during transitions within the HF cycle. In the anagen stage, adequate epithelial-mesenchymal communication is vital for matrix cell proliferation, follicular melanogenesis, and hair shaft formation. Forty-four proteins were grouped as C1, C2, and C3 based on related cellular processes, such as apoptosis, proliferation/cell survival, mitosis/ cytokinesis, and regulation of signal transduction/cell phase transition. In total, 22 proteins were up-regulated during the anagen phase (Fig 2), and 3, Annexin A1, Vimentin, and Lamin A/C, were selected for discussion.

Annexin A1 (ANXA1) is the first characterized member of the annexin superfamily, whose main property is to bind to cellular membranes in a Ca^2+^-dependent manner. ANXA1 plays an important role in membrane organization, membrane traffic, cell adhesion, migration and fusion[23]. It is associated with the cytoskeletal protein of keratinocytes, which exhibit basal-level cell staining and certain other lower layers. Accumulated evidence indicates that Annexin A1 expression is up-regulated in proliferative hepatocytes[24]. A study found ANXA1 promoted the migration of human skin fibroblast cell line WS1 cells. Wound-healing assays using ANXA1 Ac2-26 also showed that peptide was able to increase fibroblast cell migration in high glucose conditions[25]. ANXA1 links the EGF-triggered growth signal pathway with cPLA2, resulting in activation of cPLA2, a critical enzyme for growth stimulation in normal human keratinocytes (NHK). It is found that NHK treated with TGFβ showed suppressed proliferation. Meanwhile, a reduced level of cPLA2 mRNA was observed in these NHK, which means cPLA2 might play a role in epithelial cell growth inhibition[26]. During anagen, the number of mesenchymal cells in the follicular papilla and epithelial cells of hair bulbs increased rapidly [27, 28]. It was most likely through the migration of selected connective tissue sheath cells into the follicular papilla and through their proliferation[29]. It could be predicted that ANXA1 might contribute to the migration of epithelial and mesenchymal cells or may impact on hair follicle epithelial cells by interaction with cPLA2 during anagen.

Vimentin is a type III intermediate filament protein, which might be secreted from invading macrophages [30] or derived from adjacent dermal telocytes [31]. It is usually found in various non-epithelial cells. It has been shown that vimentin can also be expressed in epithelial to mesenchymal transition (EMT), a critical event in the induction of cell motility [32]. Besides, it plays a role in repair functions in wound healing [33]. Vimentin-deficient mice exhibited impaired wound healing due to defects in fibroblast migration [34]. According to a previously published article, vimentin was confirmed to be a marker of mesenchymal[35]. Our data showed that the trend of vimentin expression was consistent with the significantly increased number of mesenchymal cells in anagen.

Humans and mice share conserved A-type and B-type lamin proteins, both of which are major structural components of the inner nuclear lamina[36]. The LMNA gene encodes the A-type lamins, including the lamin A, lamin AΔ10, and lamin C proteins[37]. Mutations in A- and B-type lamin genes lead to major phenotypes reside in the skin, including alopecia, loss of subcutaneous fat, and a general atrophic condition of the skin and its appendages[38]. It has been previously described that the lamin A/C proteins were strongly expressed in mouse epidermis at embryonic day 15–17, as well as postnatally and in the adult mouse [39]. Strong expression of lamins A/C and B are found in the basal cells of the epidermis, the outer root sheath, and the dermal papilla, in all stages of the hair cycle. Also, medium expression of lamin A/C was seen in inner root sheath in anagen and catagen phase. However, lower expression of both lamins A/C and B was found in suprabasal cells of the epidermis, in the hypodermis, and in the bulb of catagen follicles[40].

There have been few reports regarding the participation of the three proteins considered herein in hair cycling. Western blotting demonstrated that these three proteins were dynamically up-regulated in the anagen stage, implying that they might facilitate hair regulation by promoting migration, mitosis, cellular homeostasis and gene regulation. Nevertheless, the specific underlying mechanisms had remained unclear.

It is well known that the catagen phase is a highly controlled process of coordinated cell differentiation and apoptosis[41]. Some studies suggest that hair-follicle apoptosis involves two different types of behaviors. One such behavior is the terminal differentiation of follicular epithelial cells in anagen hair. The other behavior eliminates the distinct portion of epithelial components in catagen hair[42]. It is recognized that TGF-β_2_ induces the up-regulation of caspase-9 and caspase-3 as well as activates the intrinsic caspase network during the catagen stage[43]. Another study found that TNFα contributes to the activation of intracellular target NF-*k*B, which induces anagen-catagen transitions[44]. The balance between apoptosis and cell survival is crucial for catagen transformation. The present bioinformatic analysis indicates that, in total, 19 differentially expressed proteins are involved in apoptosis. Seven proteins were grouped into C1 and C3 based on their expression patterns, which are consistent with their tendencies toward cell apoptosis occurring during the telogen and catagen phases. However, the mechanism governing apoptosis has not been fully characterized.

Recent studies have determined the presence of induced pluripotent stem (iPS) cells in patient biopsies for a wide variety of degenerative disorders[45]. In the mouse experiment model, plucking induces a short wound-healing response after depilation, similar to observations when hair is plucked from patients as a noninvasive way to obtain cells for reprogramming, and studies have shown that mouse DP cells can be more readily reprogrammed into iPS cells than most other cell types[46]. Furthermore, SOX_2_-positive DP cells and dermal sheath cells may have the ability to induce the origination of skin-derived progenitor cells (SKPs)[47]. Therefore, it is necessary to determine the factors responsible for reprogramming in order to use them in therapeutic applications to treat hair diseases.

Our proteome analysis included many uncharacterized proteins as well as some pluripotency marker proteins. In particular, translationally controlled tumor protein (Tpt1) may be an appropriate candidate for studying the mechanism(s) behind reprogramming. Tpt1 is involved in C2 and C3 up-regulation during the anagen and catagen phases. We speculate that Tpt1 may play an important role in activating transcription or may very well facilitate the first step in somatic cell reprogramming[48]. New candidate proteins that are involved in reprogramming may be discovered, facilitating therapeutic cloning applications.

Proteins with extreme p*I* values, less abundant proteins, very acidic or basic, very large or small proteins[49] and membrane-associated proteins can be very difficult to analyze. Integral membrane proteins and other transmembrane signaling molecules, such as FGF18, TGFb2 and GFG5, were hardly identified, which may caused by solubility constraints, difficulties encountered during extraction[50] and low concentration[51]. It is also hard to differentiate the different proteins with the same or similar M_r_ and p*I* values. In addition, we cannot rule out the possibility that a subset of proteins in the murine skin proteome undergo Avertin-induced change. To characterize proteins with these properties, supplementary purication techniques may help.

In summary, proteomics data were employed to construct a profile of protein expression throughout the hair cycle. Using bioinformatics and cluster analyses, biological networks impacted by certain proteins have been explained. These data may significantly benefit our understanding of the critical biological processes that occur during the hair cycle.

## Supporting Information

S1 Table Identified proteins. Spot ID, protein name, EntezGene ID, isoelectric point (p*I*) value, matched peptides/total peptides submitted, sequence coverage, RMS error.
(DOC)

S2 Table Results of Gene Ontology (GO) enrichment analysis. Differentially abundant proteins were classified based on the biological process, molecular function, and cellular component.
(XLSX)

S1 File The raw, unadjusted, uncropped blots and gels whose cropped versions are present in Fig 5. (ZIP)
