# Supplementary material for: Differential Expression of Proteins Associated with the Hair Follicle Cycle - Proteomics and Bioinformatics Analyses
Source: PLoS One. 2016 Jan 11;11(1):e0146791. doi: 10.1371/journal.pone.0146791 (PMC4709225; doi:10.1371/journal.pone.0146791)
Supplement: S1 Table — Spot ID, protein name, EntezGene ID, isoelectric point (pI) value, matched peptides/total peptides submitted, sequence coverage, RMS error. (DOC) [file pone.0146791.s001.doc]

| Spot ID | Protein Name | EntezGene ID | pI value | PMF | | |
| --- | --- | --- | --- | --- | --- | --- |
|  |  |  |  | Matched peptides /total peptides submitted | Coverage | RMS error (ppm) |
| 209 | Pvalb Parvalbumin alpha | 19293 | 5.02 | 11/36 | 54% | 8 |
| 324 | EG636127 12 kDa protein | 636127 | 6.9 | 7/82 | 40% | 39 |
| 492 | Sod1 Superoxide dismutase | 20655 | 6.02 | 7/35 | 41% | 10 |
| 625 | Mylpf Myosin regulatory light chain 2, skeletal muscle isoform | 17907 | 4.82 | 20/89 | 72% | 9 |
| 720 | LOC100048430;Tpt1;LOC100043703 Translationally-controlled tumor protein | 22070 | 4.84 | 10/40 | 32% | 11 |
| 1067 | LOC100048430;Tpt1;LOC100043703 Translationally-controlled tumor protein | 22070 | 4.84 | 8/37 | 32% | 15 |
| 1090 | 8430432A02Rik hypothetical protein LOC71524 | 71524 | 11.65 | 4/5 | 14% | 29 |
| 1107 | Tpm1 28 kDa protein | 22003 | 4.75 | 10/27 | 36% | 11 |
| 1119 | Apoa1 apolipoprotein A-I | 11806 | 5.51 | 19/44 | 46% | 22 |
| 1169 | LOC100042025 similar to Glyceraldehyde-3-phosphate dehydrogenase (GAPDH) | 100042025 | 8.21 | 6/14 | 14% | 12 |
| 1171 | Hspb1 Isoform B of Heat shock protein beta-1 | 15507 | 6.45 | 13/65 | 62% | 4 |
| 1270 | Anxa1 Annexin A1 | 16952 | 6.97 | 31/91 | 72% | 14 |
| 1270 | Akr1b3 Aldose reductase | 11677 | 6.71 | 12/91 | 37% | 15 |
| 1293 | Ppa1 Inorganic pyrophosphatase | 67895 | 5.37 | 14/69 | 40% | 14 |
| 1343 | Anxa8 Annexin A8 | 11752 | 5.56 |  | 3% | 6 |
|  | Anxa8 Annexin A8 | 11752 | 5.68 | 14/66 | 40% | 26 |
| 1353 | Glrx3 Glutaredoxin-3 | 30926 | 5.42 | 13/67 | 39% | 16 |
| 1366 | Ndufa10 NADH dehydrogenase [ubiquinone] 1 alpha subcomplex subunit 10, mit | 67273 | 7.63 | 13/51 | 29% | 24 |
| 1376 | Capza1 F-actin-capping protein subunit alpha-1 | 12340 | 5.34 | 9/61 | 39% | 9 |
|  | Capza2 F-actin-capping protein subunit alpha-2 | 12343 | 5.57 |  | 3% | 2 |
| Spot ID | Protein Name | EntezGene ID | pI value | PMF | | |
|  |  |  |  | Matched peptides /total peptides submitted | Coverage | RMS error (ppm) |
| 1388 | Pdhb Pyruvate dehydrogenase E1 component subunit beta, mitochondrial precu | 68263 | 6.41 | 16/62 | 37% | 11 |
| 1428 | Acads Acyl-Coenzyme A dehydrogenase, short chain | 11409 | 8.68 | 17/55 | 32% | 14 |
|  | Acads Short-chain specific acyl-CoA dehydrogenase, mitochondrial precursor | 11409 | 8.96 |  | 2% | 2 |
| 1445 | Serpinb1a Leukocyte elastase inhibitor A | 66222 | 5.85 | 28/69 | 53% | 18 |
| 1482 | Nans N-acetylneuraminic acid synthase | 94181 | 6.61 | 9/39 | 26% | 8 |
| 1513 | Tardbp TAR DNA-binding protein 43 | 230908 | 6.26 | 13/41 | 21% | 14 |
| 1580 | Acot1 Acyl-coenzyme A thioesterase 1 | 26897 | 6.12 | 7/23 | 14% | 37 |
| 1600 | Uqcrc2 Cytochrome b-c1 complex subunit 2, mitochondrial precursor | 67003 | 9.26 | 10/43 | 21% | 31 |
| 1683 | 2310057M21Rik Uncharacterized protein C10orf88 homolog | 68277 | 6.03 | 13/96 | 23% | 52 |
| 1714 | Tufm Isoform 1 of Elongation factor Tu, mitochondrial precursor | 233870 | 7.23 | 23/60 | 42% | 8 |
| 1742 | Umps Uridine 5'-monophosphate synthase | 22247 | 6.17 | 19/83 | 26% | 13 |
| 1749 | Vim Vimentin | 22352 | 5.06 | 33/91 | 60% | 14 |
| 1768 | Camk2b Calcium/calmodulin-dependent protein kinase II, beta | 12323 | 6.87 | 11/62 | 22% | 42 |
| 1774 | Dpysl2 Dihydropyrimidinase-related protein 2 | 12934 | 5.95 | 24/40 | 38% | 17 |
| 1827 | Lmna Isoform C of Lamin-A/C | 16905 | 6.37 | 36/81 | 53% | 13 |
| 1850 | Acadvl Very long-chain specific acyl-CoA dehydrogenase, mitochondrial prec | 11370 | 8.91 | 14/41 | 26% | 20 |
| 1967 | Lmna Isoform A of Lamin-A/C | 16905 | 6.54 | 34/67 | 46% | 12 |
| 1974 | Lmna Isoform A of Lamin-A/C | 16907 | 6.54 | 37/80 | 48% | 12 |
| 1993 | Ehhadh Enoyl-Coenzyme A, hydratase/3-hydroxyacyl Coenzyme A dehydrogenase | 74147 | 9.22 | 34/56 | 45% | 13 |
| 2225 | Vav2 Vav2 protein | 22325 | 6.85 | 16/93 | 20% | 46 |
| Spot ID | Protein Name | EntezGene ID | pI value | PMF | | |
|  |  |  |  | Matched peptides /total peptides submitted | Coverage | RMS error (ppm) |
| 2251 | Usp37 Isoform 2 of Ubiquitin carboxyl-terminal hydrolase 37 | 319651 | 5.74 | 16/76 | 24% | 46 |
| 2321 | Atp2a1 Sarcoplasmic/endoplasmic reticulum calcium ATPase 1 | 11937 | 5.13 | 23/80 | 19% | 30 |
| 2567 | Trf Serotransferrin precursor | 22041 | 6.94 | 17/70 | 27% | 19 |
|  | LOC634257 hypothetical protein isoform 1 |  | 12.05 | 12/70 | 38% | 45 |
| 2813 | Cps1 Carbamoyl-phosphate synthase [ammonia], mitochondrial precursor | 227231 | 6.48 | 14/28 | 8% | 21 |
| 2924 | Ankrd26 ankyrin repeat domain 26 | 232339 | 5.69 | 27/77 | 17% | 40 |
| 2941 | Dsp desmoplakin isoform 1 | 109620 | 6.43 | 29/60 | 9% | 54 |
| 3127 | Dsp desmoplakin isoform 1 | 109620 | 6.43 | 28/59 | 9% | 49 |
| 3224 | Macf1 Isoform 1 of Microtubule-actin cross-linking factor 1 | 11426 | 5.34 | 41/74 | 7% | 43 |
